# Imatinib Mesylate Exerts Anti-Proliferative Effects on Osteosarcoma Cells and Inhibits the Tumour Growth in Immunocompetent Murine Models

**DOI:** 10.1371/journal.pone.0090795

**Published:** 2014-03-05

**Authors:** Bérengère Gobin, Gatien Moriceau, Benjamin Ory, Céline Charrier, Régis Brion, Frederic Blanchard, Françoise Redini, Dominique Heymann

**Affiliations:** 1 INSERM, UMR 957, Nantes, France; 2 Université de Nantes, Nantes Atlantique Universités, Physiopathologie de la Résorption Osseuse et Thérapie des Tumeurs Osseuses Primitives, Nantes, France; 3 Equipe LIGUE Nationale Contre le Cancer 2012, Nantes, France; 4 CHU de Nantes, Nantes, France; Utrecht University, Netherlands

## Abstract

Osteosarcoma is the most common primary malignant bone tumour characterized by osteoid production and/or osteolytic lesions of bone. A lack of response to chemotherapeutic treatments shows the importance of exploring new therapeutic methods. Imatinib mesylate (Gleevec, Novartis Pharma), a tyrosine kinase inhibitor, was originally developed for the treatment of chronic myeloid leukemia. Several studies revealed that imatinib mesylate inhibits osteoclast differentiation through the M-CSFR pathway and activates osteoblast differentiation through PDGFR pathway, two key cells involved in the vicious cycle controlling the tumour development. The present study investigated the *in vitro* effects of imatinib mesylate on the proliferation, apoptosis, cell cycle, and migration ability of five osteosarcoma cell lines (human: MG-63, HOS; rat: OSRGA; mice: MOS-J, POS-1). Imatinib mesylate was also assessed as a curative and preventive treatment in two syngenic osteosarcoma models: MOS-J (mixed osteoblastic/osteolytic osteosarcoma) and POS-1 (undifferentiated osteosarcoma). Imatinib mesylate exhibited a dose-dependent anti-proliferative effect in all cell lines studied. The drug induced a G0/G1 cell cycle arrest in most cell lines, except for POS-1 and HOS cells that were blocked in the S phase. In addition, imatinib mesylate induced cell death and strongly inhibited osteosarcoma cell migration. In the MOS-J osteosarcoma model, oral administration of imatinib mesylate significantly inhibited the tumour development in both preventive and curative approaches. A phospho-receptor tyrosine kinase array kit revealed that PDGFRα, among 7 other receptors (PDFGFRβ, Axl, RYK, EGFR, EphA2 and 10, IGF1R), appears as one of the main molecular targets for imatinib mesylate. In the light of the present study and the literature, it would be particularly interesting to revisit therapeutic evaluation of imatinib mesylate in osteosarcoma according to the tyrosine-kinase receptor status of patients.

## Introduction

Osteosarcoma is a rare bone tumour mainly affecting young patients (peak of incidence around 18 years old), defined by the presence of tumour cells producing an osteoid matrix [1]. The current therapeutic sequence for high-grade osteosarcoma was proposed by Rosen et al in the 1970s and is now internationally accepted [2]. This treatment is based on neo-adjuvant chemotherapy, delayed en-bloc wide resection, and adjuvant chemotherapy adapted to the histologic profile assessed on tumour tissue removed during surgery. In spite of the fact that the development of polychemotherapy has clearly improved the survival and the quality of life of patients, the 5-year event-free survival has remained at a plateau of 60–70% of patients with non-metastatic osteosarcoma for over the last 40 years [3]. Nevertheless, in the last 10 years, better knowledge of oncogenic processes in osteosarcoma has led to the development of new therapeutic approaches based on single new drugs or administered in combination with conventional chemotherapy [4].

Targeting intra-cellular signaling or metabolic pathways appear as promising therapeutic approaches. For instance, mevalonate pathway may be an interesting target in osteosarcoma. Thus, the combination of apomine and lovastatine which targets the mevalonate pathways significantly reduced tumour progression in osteosarcoma-bearing mice compared to single treatment which had no effect at the doses used [5]. The mTOR inhibitor ridaforolimus has been studied in a phase II trial of patients with advanced bone sarcomas and this study revealed improved progression-free survival in advanced sarcomas including osteosarcoma [6]. Moriceau *et al.* demonstrated recently that RAD001 (everolimus) a new oral mTOR inhibitor, inhibited osteosarcoma cell proliferation and its combination with zoledronic acid reduced tumour development in murine models of mixed osteoblastic/osteolytic or undifferentiated osteosarcoma [7]. Similarly, NVP-BEZ235, a dual pan-PI3K-mTOR inhibitor, exhibits anti-proliferative effects in a panel of osteosarcomas and showed synergistic activity with chemotherapeutic agents and with other small signaling inhibitors [8]. In contrast, targeting Src, a tyrosine kinase which activates tumour cell-motility and invasion showed interesting anti-proliferative and pro-apoptotic activity in osteosarcoma cell lines but did not exert any activity *in vivo* [9].

Imatinib mesylate (Gleevec) is an orally active tyrosine kinase inhibitor with activity against a large panel of tyrosine kinase protein including bcr/abl, c-kit, MCSF receptor (cFMS) and the PDFG receptor among others [10]. Its clinical success is demonstrated by its current use as a first-line therapy for patients with bcr-abl-positive chronic myeloid leukemia [11] and in gastrointestinal stromal cell tumours characterized by activating mutations of c-kit [12]. In addition, imatinib mesylate exerts direct effects on bone cells. It inhibits osteoclast-resorbing activity by increasing mature osteoclast apoptosis and targeting the MCF receptor [13]. Moreover, imatinib mesylate also alters osteoblast differentiation with promoting [14] or deleterious activities [15]. It seems that the drug decreases osteoblast proliferation while stimulating their activity. All published data suggest that imatinib mesylate may transiently increase osteoblast activity and subsequently induce an opposite effect [16]. Based on these observations, imatinib mesylate may be an interesting drug for bone sarcoma and especially for osteosarcoma.

The present work aimed to analyze the biological activities of imatinib mesylate in various human, mouse and rat osteosarcoma cell lines and on primary tumour growth (at the bone site) using two different syngenic models of murine osteosarcoma.

## Materials and Methods

### Ethic Statement

Mice (Elevages Janvier, France) were housed under pathogen-free conditions at the Experimental Therapy Unit (Faculty of Medicine, Nantes) in accordance with the institutional guidelines of the ethical committee and under the supervision of authorized investigators. The Institutional Animal Care and Use Committee (CEEA PdL 06) approved specifically the study (authorization number: 1280.01).

### Cells and Culture Conditions

The rat osteosarcoma OSRGA cell line established from a radio-induced osteosarcoma [17] and human HOS, MG63 cells purchased from ATCC (Promochem, France) were cultured in DMEM (Lonza, Belgium) supplemented with 10% FCS (Hyclone, USA). Murine osteosarcoma POS-1 and MOS-J cells derived from mouse spontaneous osteosarcoma were provided respectively by Dr Kamijo [18] and by Dr Shultz [19] and were cultured in RPMI with 10% FCS. Cells expressed osteoblastic markers more specifically cbfa1/Runx2, bone alkaline phosphatase, and MOS-J and OSRGA cells were able to form mineralized nodules *in vitro* (data not shown). These parameters were tested before cell implantation.

### Cell Growth, Viability

Two thousand cells per well were plated into 96-well plates and cultured for 72 h in the presence or absence of imatinib mesylate (10–40 µM). Cell growth and viability was determined by the sodium 3′[1-(phenylaminocarbonyl)-3,4-tetrazolium]-bis(4-methoxy-6-nitro)benzene sulfonic acid hydrate (XTT) cell proliferation reagent assay kit (Roche Molecular Biomedicals, Germany). After the culture period and addition of the XTT reagent, the absorbance was then determined at 490 nm. Caspase activity was assessed on 10 µl of total cell lysates using the kit CaspACE Assay System (Promega, USA), following the manufacturer’s recommendations. Results were expressed in arbitrary units and corrected for protein content quantified using the BCA [bicinchominic acid+copper(II) sulfate] test (Pierce Chemical Co.). In some experiments, cells were treated with 20 µM of imatinib mesylate in the presence or the absence of 50 µM of caspase inhibitor *N*-benzyloxylcarbonyl-Val-Ala-Asp (OCH3) fluoromethyketone (Z-VAD-FMK)(Promega, France). Cells treated with 100 nM of Staurosporin for 24 hours were used as a positive control. Imatinib mesylate was provided by Pharma Novartis AG (Switzerland).

### Time-lapse Microscopy and Wound Healing Assay

Osteosarcoma cells were cultured at 5×10^3^cells/mm^2^ (24-multiwell plate) in the presence or absence of imatinib mesylate (10–25 µM). Phase-contrast photos were taken every 10 minutes for 72 hours through a Leica microscope using a X10 objective [Leica DMI 6000B (Wetzlar, Germany) coupled with a Coolsnap HQ2 video camera (Roper Scientific, Evry, France)], then Quick Time movies were edited with the Metamorph 7.5 software (Roper Scientific). Cell divisions and dead cells were then manually scored in each field of observation in a time-dependent manner. The cells accompanied by extensive plasma membrane blebbing were considered as apoptotic cells. For wound healing assay, osteosarcoma cell monolayers were damaged by scraping with a micropipette tip then incubated for 24 hours in the presence of 4 µg/mL of mitomycin with or without imatinib mesylate (10–40 µg/mL). The extent of cell migration into the wounded area was analyzed by comparing microphotographs after 24 hours of treatment. Each condition was performed in duplicate.

### Cell Cycle Analysis

Sub-confluent OSRGA, MG63, POS-1 or MOS-J cells were incubated with or without imatinib mesylate (10–25 µM) for 24 hours to 72 hours. After the treatment period, cells were removed from culture dishes by trypsinization, washed twice in PBS, and incubated in PBS containing 0.12% of Triton X-100, 0.12 mmol/L of EDTA, and 100 µg/mL of DNase-free RNase A (Sigma). Then, 50 µg/ml of propidium iodide were added for 20 minutes at 4°C in the dark. Cell cycle distribution was studied by flow cytometry (Cytomics FC500, Beckman Coulter,France), based on 2 N and 4 N DNA content and was analyzed by DNA Cell Cycle Analysis Software (Phoenix Flow System, USA).

### Western Blot Analysis

Two hundred thousand osteosarcoma cells were cultured in 6-multiwell plates and treated with imatinib mesylate (15 or 25 µM) for 12 hours or 24 hours and then lysed in radioimmunoprecipitation assay (RIPA) buffer (150 mM Tris-NaCl, 5% Tris, pH 7.4, 1% NP-40, 0.25% Na deoxycholate, 1 mM Na_3_VO_4_, 0.5 mM PMSF, 10 mg/ml leupeptin, 10 mg/ml aprotinin). In some experiments, serum-starved osteosarcoma cells were treated with 50 ng/mL PDGF-BB (R&D System) for 5 minutes in the presence of the absence of 25 µM imatinib mesylate. Cell lysates were cleared of debris by centrifugation at 12,000 g for 15 minutes. Protein concentration was determined using the BCA kit (Pierce Chemical). Twenty µg of total cell lysate proteins were run on 10% of sodium dodecyl sulfate-poly-acrylamide gel electrophoresis (SDS-PAGE) and electrophoretically transferred to an Immobilon- P membrane (Millipore, Bedford, MA, USA). The membrane was blotted with antibodies to p-mTOR (Ser 2448), p-AKT (Ser 473), p-ERK1/2 (Thr202/Tyr204**)**, pPDGFRα (Tyr 754), p-PDGFRβ (Tyr 751), p-ERK1/2 (Thr202/Tyr204), actin or the total forms of protein described above (Table 1) in PBS, 0.05% Tween 20, and 3% bovine serum albumin (BSA). The membrane was washed and probed with the secondary antibody coupled to horseradish peroxidase. Antibody binding was visualized with the enhanced chemiluminescence system (ECL Kit; Roche Molecular Biomedicals). For quantification, the emitted glow was acquired with a CCD camera (Syngene, Cambridge, UK) and analyzed with the GeneTools software (Syngene).

**Table 1:** Primary antibodies used for cell signaling analysis.

Antibodies and origin	Phosphorylated residue	Species	Dilution
P-PDGFRα	Tyr 754	Rabbit	1/1000
PDGFRα		Rabbit	1/1000
P-PDGFRβ	Tyr 751	Rabbit	1/1000
PDGFRβ		Rabbit	1/1000
P-mTOR	Ser 2448	Rabbit	1/1000
mTOR		Rabbit	1/1000
P-AKT	Ser 473	Rabbit	1/1000
AKT		Rabbit	1/1000
P-ERK1/2	Thr202/Tyr204	Rabbit	1/1000
ERK1/2		Rabbit	1/1000
Actin	NA	Rabbit	1/20000

### Phospho-Receptor Tyrosine Kinase (RTK) Array

A commercial antibody-based protein microarray designed to detect 49 human Phospho-receptor tyrosine kinase array kit (R&D System, UK) was used to identify the molecular targets of imatinib mesylate. Experiments were carried out following manufacturer’s instructions. Briefly, array membranes were blocked with the saturated buffer for 1 hour and then incubated for 2 hours with HOS osteosarcoma cell lysates treated or not with 50 µM imatinib mesylate for 45 minutes. After washing, the membranes were incubated overnight with a specific peroxidase-labeled streptavidin anti-pTyr antibody. The membranes were then washed, and detection of immunoreactive spots was revealed by chemiluminescence detection system (GE Healthcare, France). The intensity of the chemiluminescence was acquired using the ChemiDoc XRS^+^ system (BioRad, France). The intensities of the various spot were determined using Gene Tools software.

### RNA Extraction and Semi-quantitative Reverse Transcription-polymerase Chain Reaction (RT-PCR)

Total RNA was isolated from cultured MG63, HOS, MOS-J, POS-1 and OSRGA osteosarcoma cells using the TRIzol reagent (Invitrogen, France). First, RNA was reverse-transcribed (RT), using 400 U of MMLV-RT from Invitrogen, then 2 µl of the RT reaction mixture were subjected to PCR using upstream and downstream primers to determine the expression of human, mouse and rat PDGFRα/β [30 pmol each, Table 2] and 0.25 µl of 5 U/µl Taq polymerase (Eurobio, France). PCR products were analyzed in 1% agarose gels, stained with gel RED and photographed. Relative expression of the PDGFRα and PGDFRβ genes were compared to the 18S signal and band densities were measured using the Image Quant computer software program. After the number of PCR cycles was increased, a plot was done for each sample, and the cycle value corresponding to the mid of the linear part of the amplification curve were used to analyse the expression of the corresponding gene.

**Table 2:** Primer sequences for RT-PCR experiments.

Gene		Sequences		Size (bp)
Human	PDGFRα	sens	AAG ATA ATG ACT CAC CTG GGG	495
		anti-sens	AGC CAA AAA CTC CAT TCC TCG	
	PDGFRβ	sens	AGG TGA TTG AGT CTG TGA GC	630
		anti-sens	TAT CGT AAG GGG CCA TGT AG	
Mouse	PDGFRα	sens	AAG ATA ATG ACT CAC CTG GGG	495
		anti-sens	AGC CAA AAA CTC CAT TCC TCG	
	PDGFRβ	sens	AGG TGA TTG AGT CTG TGA GC	630
		anti-sens	TAT CGT AAG GGG CCA TGT AG	
Rat	PDGFRα	sens	CAG GTC TAG TGA GAA GCA AGC TC	382
		anti-sens	CGA TCT CTG GAT GTC GGA GTA	
	PDGFRβ	sens	GGT ACG TGT GAA GGT GTC AGA AG	275
		anti-sens	GGC TCT CCT CCT TGG AAC TAT T	
House keeping gene	18S	sens	TCA AGA ACG AAA GTC GGA GGT TCG	462
		anti-sens	TTA TTG CTC AAT CTC GGG TGG CTG	

### Mixed Osteoblastic/Osteolytic Osteosarcoma Model

Four-week-old male C57BL/6J mice were anesthetized by inhalation of a combination Isoflurane/air associated with an i.m. injection of Buprenorphine (Temgésic, Schering-Plough) before an i.m. injection of 4×10^6^ MOS-J cells. Tumours appeared in contact with the tibia approximately 8 days later and led to osteoblastic lesions reproducing the osteoblastic form of human osteosarcoma associated with osteolytic foci [7]. Three groups of eight mice each were assigned as controls (placebo by daily oral administration) and imatinib mesylate (50 and 100 mg/kg, daily oral administration) groups. Two types of experiments were carried out: (i) treatment started one day after tumour cell implantation named “preventive treatment”, (ii) treatment started when tumours were palpable (7–10 days) named “curative treatment”.

### Undifferentiated Osteosarcoma Model

Four-week-old male C3H/He mice were anesthetized as previously described before s.c. inoculation of 2×10^6^ POS-1 cells in the hind footpad of the mice. Under these conditions, mice develop a primary tumour at the site of injection in 3 weeks that can be transplanted to mice of the same strain as a small fragment (2×2×2 mm) in close contact with the tibia. For this purpose, the periostum of the diaphysis was opened and resected along a length of 5 mm, and the underlying bone was intact. The osteosarcoma fragment was placed contiguous to the exposed bone surface without the periostum, and the cutaneous and muscular wounds were sutured. Tumours appeared at the graft site approximately 8 days later, associated with the development of pulmonary metastases in a 3-week period. The tumours that developed in contact with the femora lead to osteolytic lesions that reproduced the osteolytic form of human osteosarcoma (7, 20). Two groups of eight mice each were assigned as controls (placebo by daily oral administration) and 100 mg/kg imatinib mesylate (daily oral administration) groups. The treatment started one day after tumour cell implantation.

For both models, the tumour volumes (V) were calculated from the measurement of two perpendicular diameters using a caliper according to the following formula: V = 0.5×L×S^2^, where L and S represent respectively, the largest and smallest perpendicular tumour diameters. Treatment continued until each animal showed signs of morbidity including cachexia or respiratory distress, at which point they were sacrificed by cervical dislocation. Analysis of architectural parameters was done using high-resolution X-ray micro-computed tomography (CT) (SkyScan-1076). Relative volume (BV/TV) of the tibia [total bone (cortical+trabecular) or trabecular bone] was quantified at necropsy on a 3.2 cm length area located between superior metaphysis and diaphysis. Radiographs were taken at the same time (PLANMED Sophie apparatus, Finland). Each experiment was repeated twice and was reproducible. Only one set of experiments was shown.

### Statistical Analysis

Each experiment was repeated independently three times in triplicate. The mean±SD was calculated for all conditions and compared by ANOVA followed by Bonferroni *post hoc test.* Differences relative to a probability of two-tailed p<0.05 were considered significant.

## Results

### Imatinib Mesylate Exerts Anti-proliferative Effects on Human, Mouse and Rat Osteosarcoma Cells

**Figure 1:**
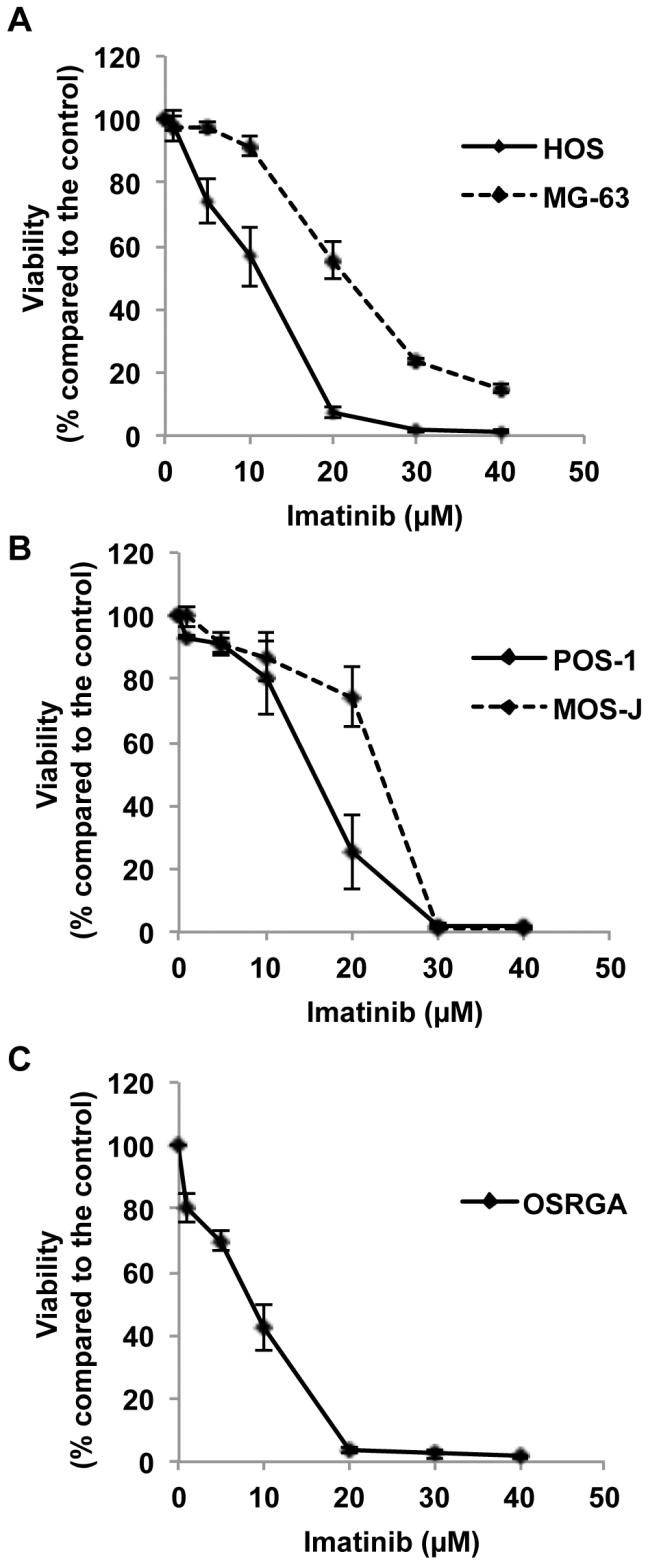
Imatinib mesylate inhibits in a dose dependent manner the osteosarcoma cells proliferation. Human (HOS, MG63) (**A**)**,** mouse **(**POS-1, MOS-J**)** (**B**) and rat (OSRGA) (**C**) osteosarcoma cell lines were treated by increasing concentration of imatinib mesylate (0.1–40 µM) for 72 hours. The number of viable cells was then determined using an XTT assay. (**D**) Table summarizing the IC50 and IC90 of each cell lines studied. Graphs represent the average values of three independent experiments performed in triplicate.

**Figure 2:**
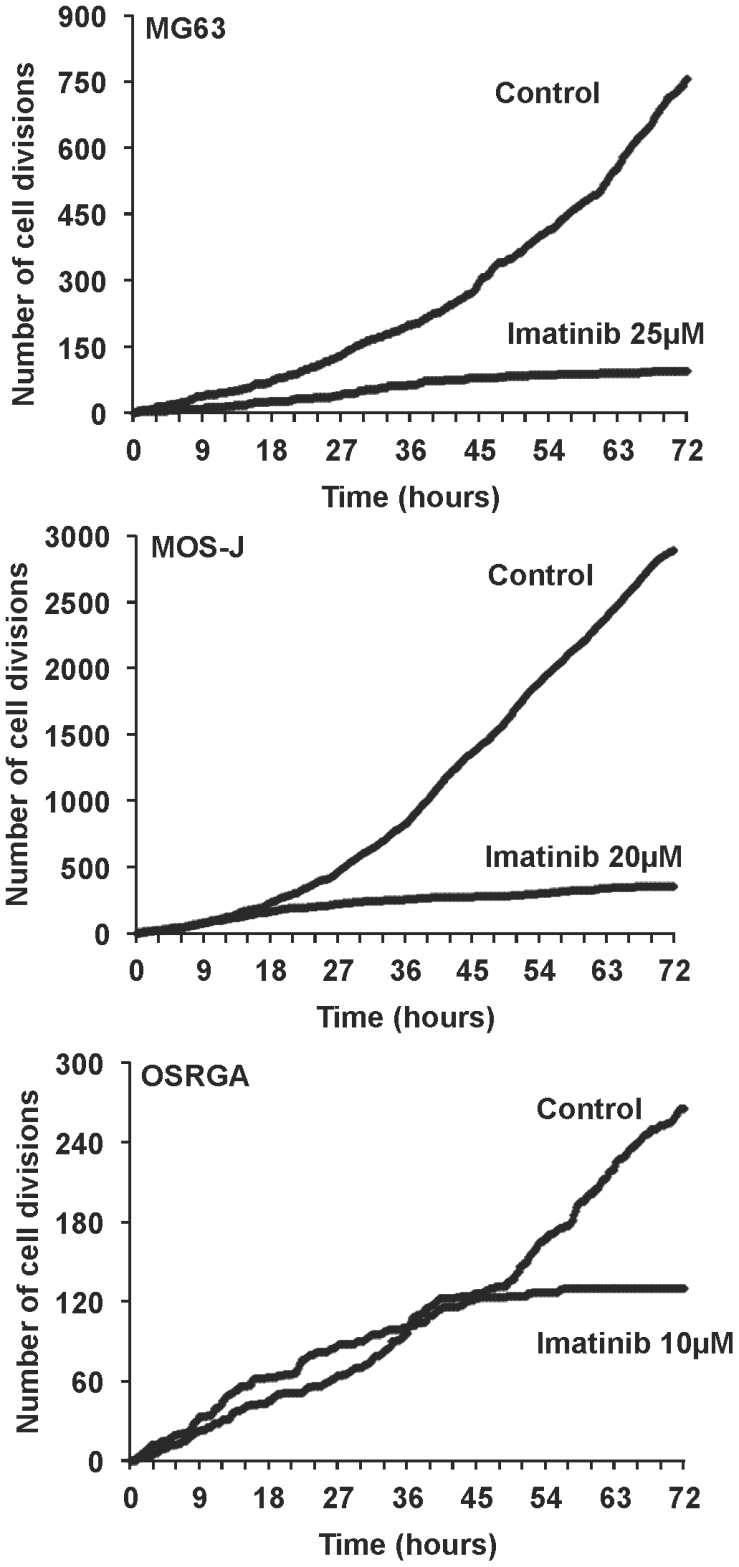
Inhibitory effect of imatinib mesylate on osteosarcoma cell mitosis. Human MG63 (**A**), mouse MOS-J (**B**) and OSRGA (**C**) osteosarcoma cells were cultured in the presence or absence of imatinib mesylate with 25 µM, 20 µM and 10 µM respectively. Phase-contrast photos were taken every 10 minutes for 72 hours and the number of cell mitosis manually scored in a time-dependent manner.

**Figure 3:**
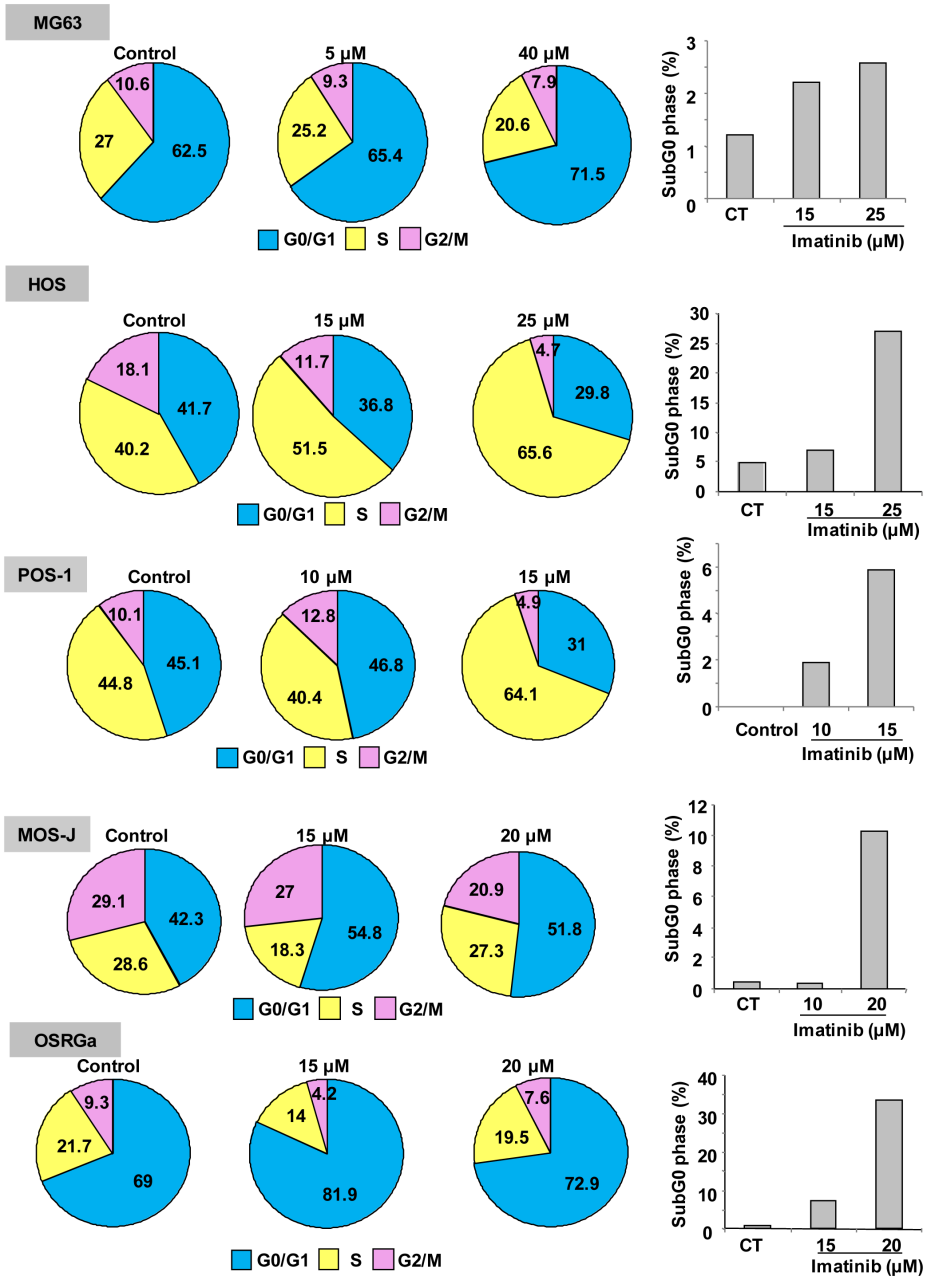
Imatinib mesylate affects osteosarcoma cell proliferation by inducing a cell cycle arrest. Cell cycle distribution of human, mouse and rat osteosarcoma cell lines treated or not with imatinib mesylated for 48 hours was analyzed by propidium iodide staining and flow cytometry. All experiments were repeated 3 times, and representative results are shown.

To determine whether imatinib mesylate is able to modulate viable osteosarcoma cell number, XTT assays have been carried out for 72 hours in the presence or absence of imatinib mesylate (1–40 µM). As shown in Figure 1, imatinib mesylate treatment of human (Figure 1A), mouse (Figure 1B) and rat (Figure 1C) osteosarcoma cells strongly reduced their viability. Thus, imatinib mesylate decreased the number of viable osteosarcoma cells in a dose-dependent manner [IC50 at 72 hours: 20 µM (MG-63), 11 µM (HOS), 23 µM (MOS-J), 15 µM (POS-1); 9 µM (OSRGA); IC90 at 72 h: 30 µM (MG-63), 19 µM (HOS), 28 µM (MOS-J), 24 µM (POS-1); 16 µM (OSRGA)] with a maximum effect at 30 µM for most of the cells assessed (Figure 1D). To determine whether the effects of imatinib mesylate on osteosarcoma cells resulted from the inhibition of cell proliferation and/or the induction of cell death, the number of cell divisions determined by time-lapse microscopy was manually counted. Time-lapse microscopy revealed that imatinib mesylate clearly markedly decreases the number of mitosis in a time- and dose-dependent manner in all cell lines assessed (Figure 2, Figure S1) (Data not shown for HOS and POS-1 cells). In addition, imatinib mesylate treatment affected the various cell cycle phases of all osteosarcoma cells compared to the untreated control (Figure 3). In MG63, MOS-J and OSRGA cells, imatinib mesylate induced a cell cyle arrest in G0/G1 phase. Indeed, the number of cells in G0/G1 phases increased from 62 to 71% for MG63 cells, from 42 to 52% for MOS-J and from 69 to 82% for OSRGA cells when treated with imatinib mesylate. The number of cells in S phase strongly increased from 40 to 65% HOS cells and from 45 to 64% for POS-1 cells when treated with the drug. Concomitantly, the cells in the apoptotic sub-G0/G1 peak also increased in all cell lines treated (Figure 3).

### Imatinib Mesylate Induces Osteosarcoma Cell Death

**Figure 4:**
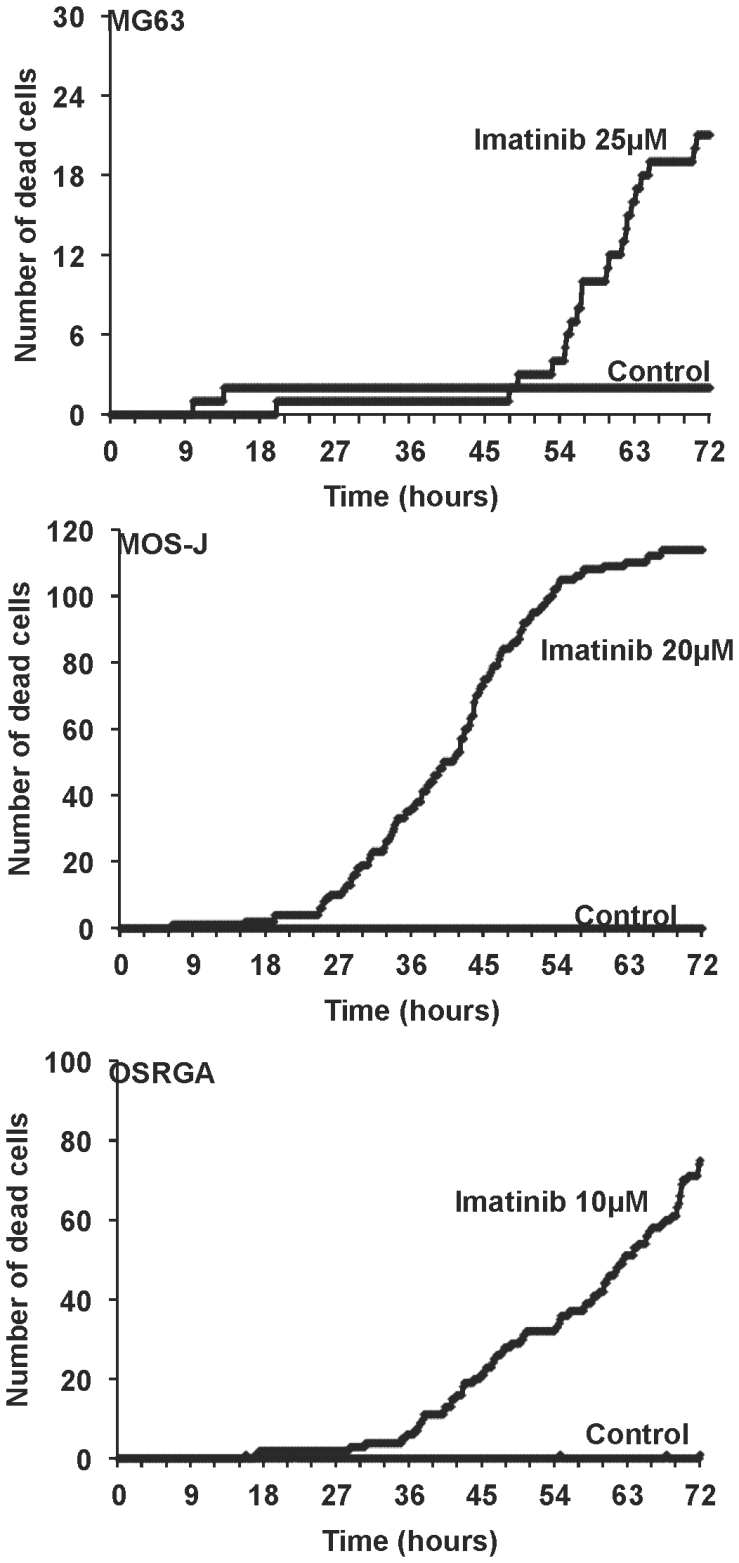
Imatinib mesylate increases osteosarcoma cell death. A kinetic of human (MG63), mouse (MOS-J) and rat (OSRGA) osteosarcoma cell death was analyzed by time-lapse microscopy in the presence or the absence of 25 µM, 20 µM or 10 µM imatinib mesylate respectively. The number of cell death was manually scored every 10 minutes until 72 hours.

**Figure 5:**
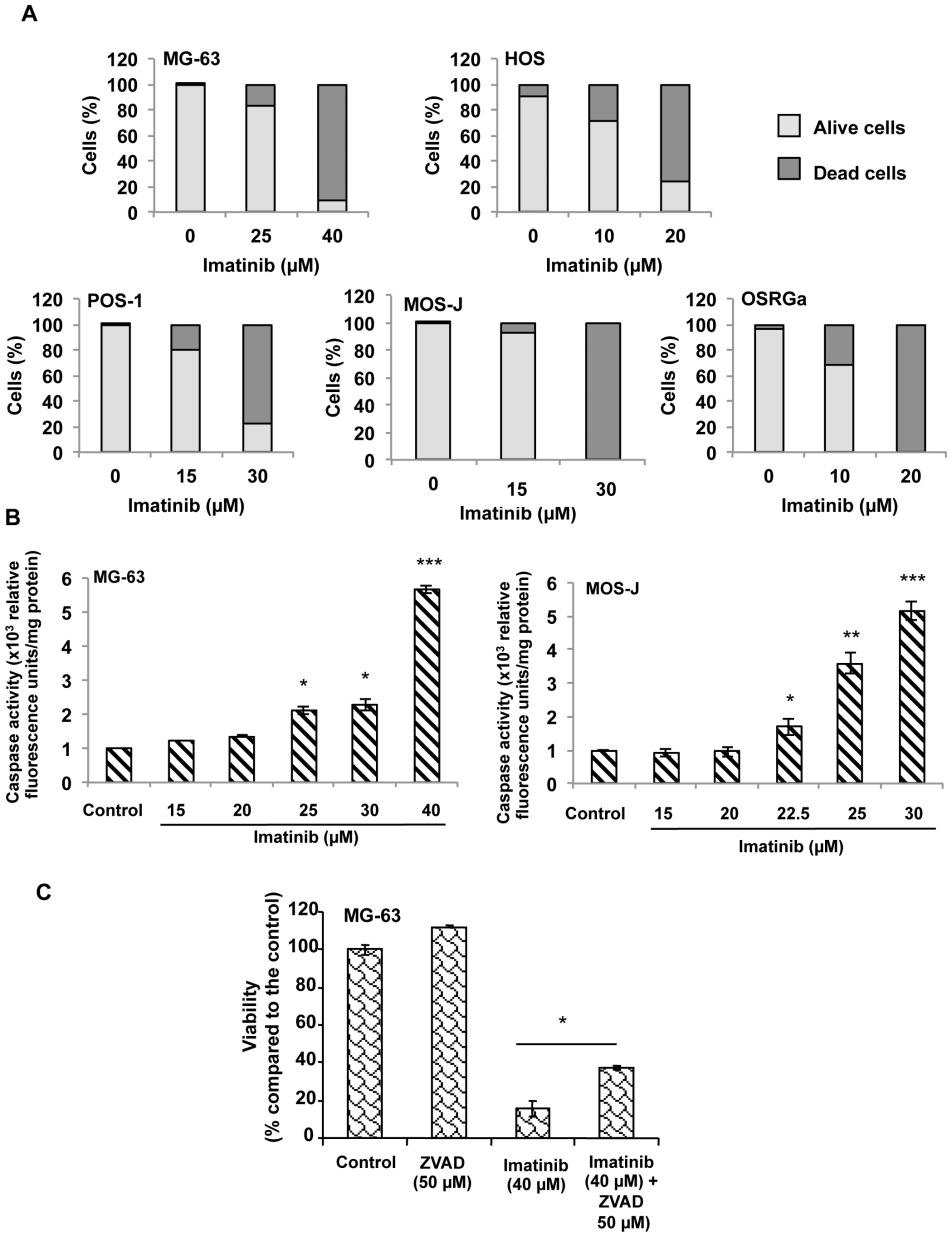
Osteosarcoma cell death induced by imatinib mesylate is partly dependent of caspase activity. (**A**) Human (MG63, HOS), mouse (POS-1, MOS-J) and rat osteosarcoma cells were cultured with or without increasing concentrations of imatinib mesylate. After 48 days of treatment, the alive and dead cells number was manually scored (from trypsinized and floating cells) after trypan blue exclusion. (**B**) Using similar culture conditions, caspase-3 activity was assessed using a kit CaspACE Assay System (Promega, USA). (**C**) On the same way, the involvement of caspase activity in cell death induced by imatinib mesylate was analyzed using the 2,3-bis(2 methoxy-4 nitro-5-sulfophenyl)2H-tetrazolium-5-carboxanilide (XTT) assay in the presence or the absence of 50 µM pan-caspase inhibitor Z-Vad-FMK(ZVAD).*p<0.05; ** p<0.01; ***p<0.001.

To determine whether the inhibitory activity of imatinib mesylate observed in osteosarcoma cell lines was associated with cell death induction, we used time-lapse microscopy to monitor the apoptotic events in human, mice and rat osteosarcoma cells treated with the drug. Time lapse analyses revealed that imatinib mesylate induced an increase of human, mice and rat osteosarcoma cell death in a dose-and time-dependant manner (Figure 4, Figure S2) (Data not shown for HOS and POS-1 cells). The number of dead/viable cells was also assessed by manual cell counting based on a trypan blue exclusion assay. Results confirmed that imatinib mesylate induced cell death of all cell lines assessed, in a dose-dependent manner (Figure 5A) associated with a significant increase of caspase activity (Figure 5 B, C). The pan-caspase inhibitor Z-Vad-FMK partly inhibited the drug-induced effects on osteosarcoma cell viability as shown for human MG63 cell line in Figure 5C (p<0.01). Overall, these data revealed that imatinib mesylate induced apoptosis of all analyzed osteosarcoma cell lines by a mechanism partly dependent of caspase activition. In addition, to its functional activity on cell death, we assessed the effect of imatinib mesylate on cell migration. As shown in Figure S3, imatinib mesylate strongly slowed down the migration of human, mouse and rat osteosarcoma cells. These data demonstrate that imatinib mesylate therefore exerts cytostatic activity on osteosarcoma.

### Imatinib Mesylate Inhibits Osteosarcoma Progression *in vivo*

**Figure 6:**
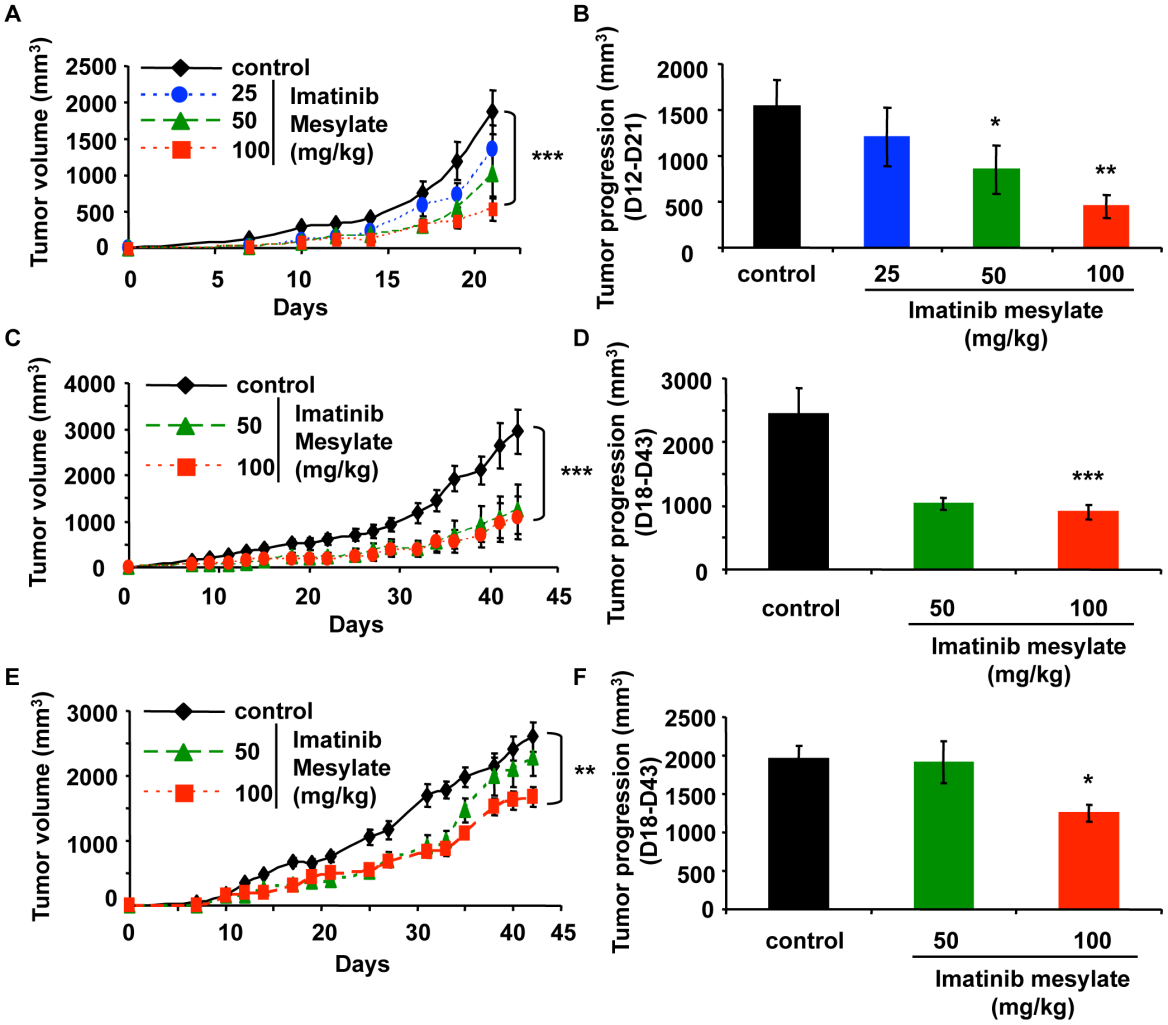
Imatinib mesylate inhibits osteosarcoma development in “preventive” and “curative” therapeutic context. Mice bearing undifferentiated POS-1 (**A, B**) or mixed osteoblastic/osteolytic MOS-J (**C–F**) osteosarcoma tumours (n = 8 per group) were assigned as control (vehicle), or imatinib mesylate (25, 50 or 100 mg/kg, daily oral administration). The treatment started 1 day after tumour cell inoculation (« preventive » treatment, **A–D**) or treatment started when tumours are palpable (7–10 days) named “curative treatment” (**E, F**). Evolution of tumour volumes (mm^3^) (A, C, E); follow-up of tumour progressions (B, D, F). * P<0.05; ** P<0.01; *** P<0.001.

In the light of potent inhibitory effects of imatinib mesylate on human, mice and rat osteosarcoma cell lines *in vitro*, we next assessed the effect of the drug on osteosarcoma tumour growth. Two pre-clinical models of mouse syngenic osteosarcoma have been used and “preventive” and “curative” treatments have been tested. The *in vivo* effects of “preventive” treatments on tumour growth were first studied in osteolytic POS-1 osteosarcoma model (Figure 6A,B). Imatinib mesylate reduced significantly in a dose-dependent manner the tumour volume compared to the control group (Figure 6A). Rate of tumour progression between days 12 and 21 was also significantly decreased in the treated group compared to the controls (1856±281 mm^3^ for control mice vs. 635±123 mm^3^ for daily 100 mg/kg imatinib mesylate treated mice; p<0.001) (Figure 6B). Similar experiments were carried out using a mixed osteoblastic/osteolytic MOS-J osteosarcoma model (Figure 6C,D). All animal treated with imatinib mesylate (n = 8 per group) exhibited a significant decrease in tumour volume compared to the control group. In a comparable manner to the POS-1 model, the studied drug reduced the rate of tumour progression at days 18 and 43 (Figure 6D). To mimic the clinical context where tumour was mainly diagnosed in patients when the tumour mass was detectable, we assessed the “curative” effect of imatinib mesylate animal treatment started when MOS-J tumours were palpable (7–10 days) (Figure 6E,F). Although imatinib mesylate was substantially less effective in the curative regimen than in the preventive one, hundred mg/kg of compound delayed the increase in tumour volume consecutively to its first administration (Figure 6E). The relative tumour progression calculated between day 18 and day 43 confirmed the significant inhibitory effect of imatinib mesylate on osteosarcoma growth (1878±171 mm^3^ for control mice vs. 1245±110 mm^3^ for 100 mg/kg imatinib mesylate treated mice; p<0.001) (Figure 6F). We then analyzed the impact of imatinib mesylate on bone microarchitecture parameters and did not observe significant effect of the drug on BV/TV and the other bone parameters (data not shown).

### PDGFRα is a Key Target of Imatinib Mesylate in Osteosarcoma

**Figure 7:**
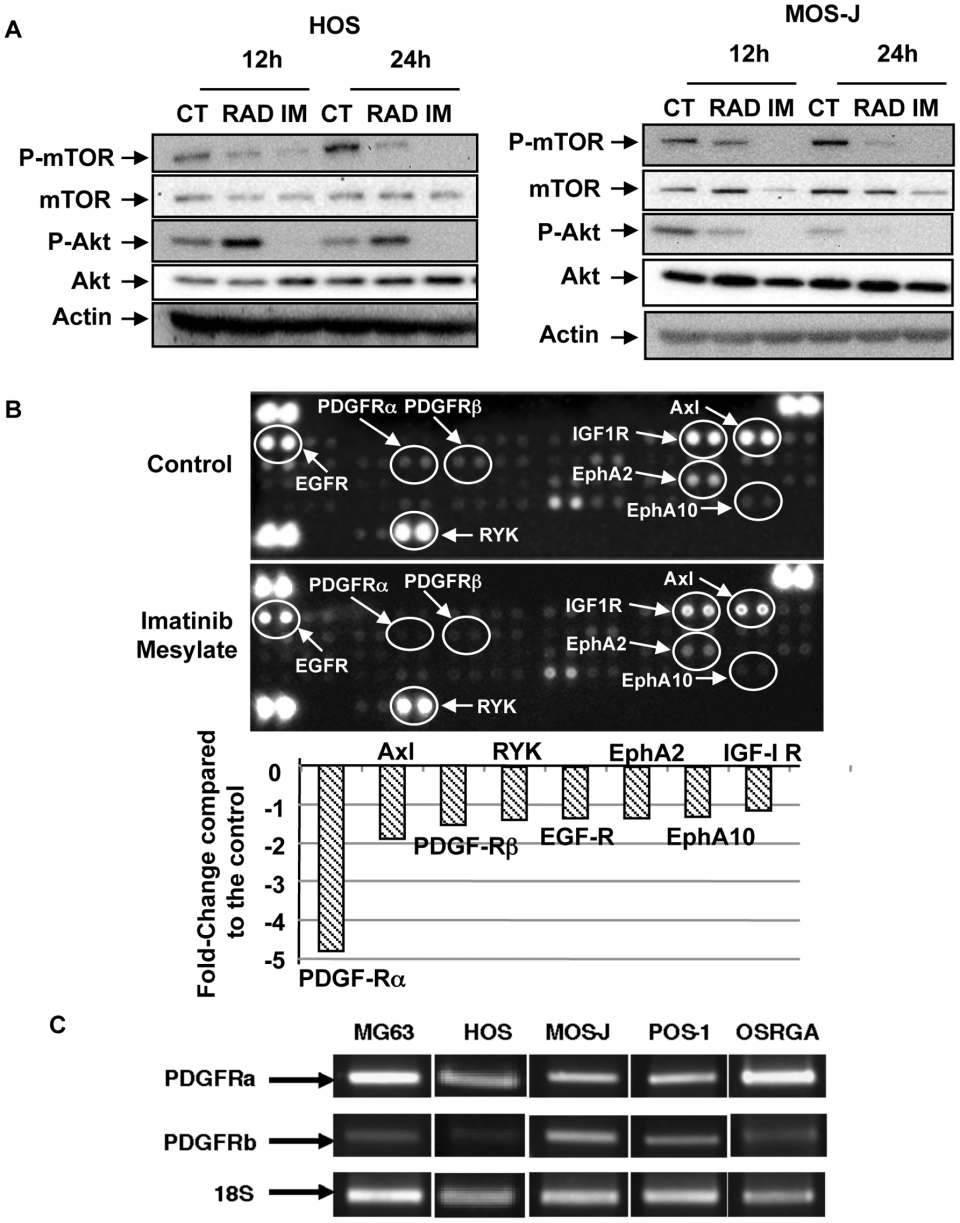
Imatinib mesylate inhibits AKT/mTOR signaling pathway in osteosarcoma cells and activates ERK1/2 phosphorylation: PDGFRα, a key target of osteosarcoma cells. Human, mouse and rat osteosarcoma were treated with various doses of imatinib mesylate to analyse the effects of the drug on intra-cellular signaling pathways. (**A**) Imatinib mesylate inhibits mTOR and Akt phosphorylation in human HOS and mouse MOS-J cells. RAD001 named everolimus (RAD), a mTOR inhibitor was used as a positive control. (**B**) Human Phospho-receptor tyrosine kinase array Kit was used to identify the molecular targets of imatinib mesylate. (C) Expression of PDGFRα and PDGFRβ analyzed in human, mouse and rat osteosarcoma cells by semi-quantitative RT-PCR.

**Figure 8:**
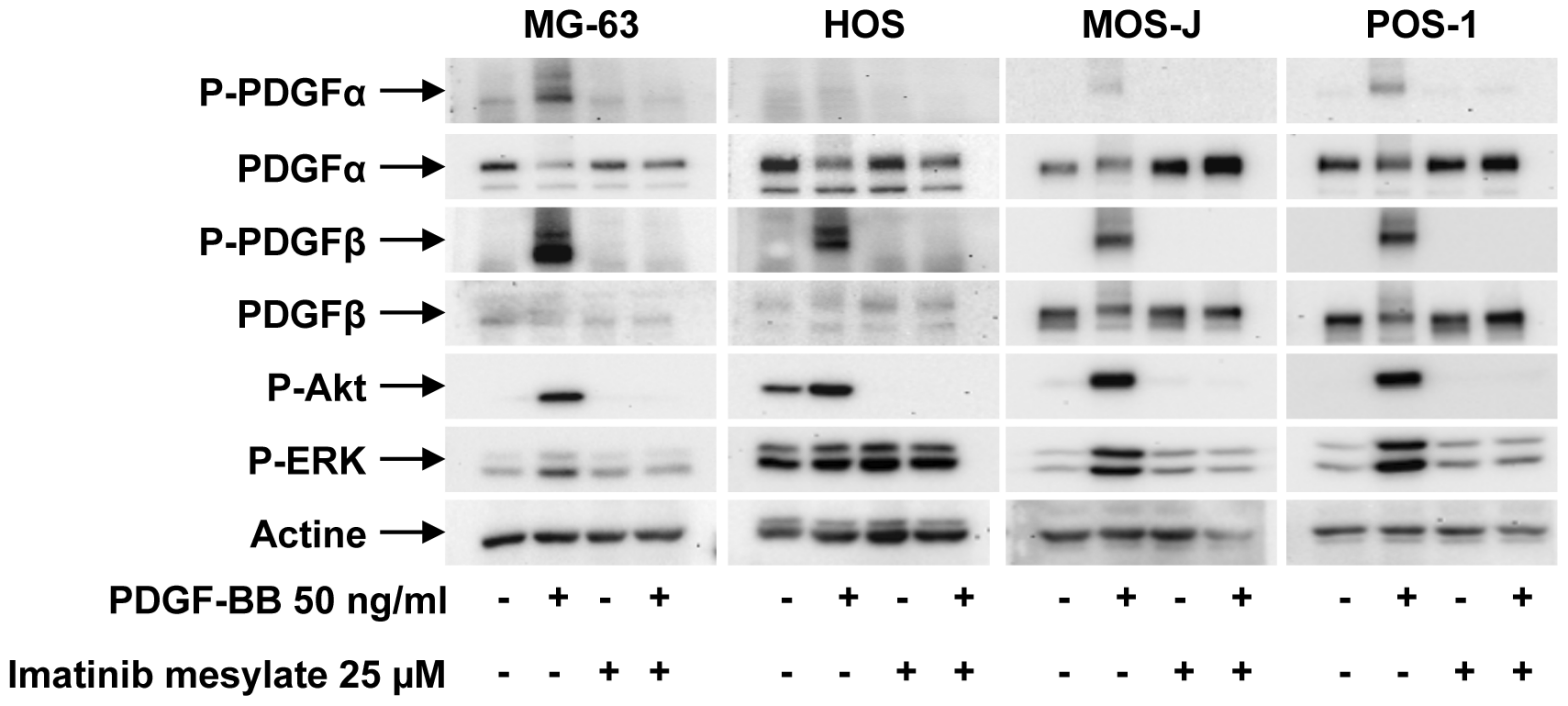
Imatinib mesylate inhibits the PDGF-BB induced signalling pathways. Human, mouse and rat osteosarcoma were treated with 50/mL of PDGF-BB for 5 minutes in the presence of the absence of 25 µM of imatinib mesylate. PDGFRα, PDGFRβ, Akt, ERK1/2 phoshorylations were analyzed by Western blot compared to the levels of total forms of proteins and the levels of actin.

The functional activity of imatinib mesylate was confirmed by western blot analysis (Figure 7). Similarly to RAD001, imatinib mesylate inhibit mTOR pathway in human and murine osteosarcoma cells (Figure 7A). In contrast to RAD001 for which a feedback loop takes place in HOS cells (7), imatinib mesylate markedly abolished Akt phosphorylation (Figure 7A). This therapeutic association did not reveal any significant synergistic or additive effect on osteosarcoma cells compared to single treatments (data not shown). To better identify the main targets of imatinib mesylate in osteosarcoma, phospho-RTK arrays were performed in the human HOS cell line. Figure 7B clearly shows several tyrosine receptor kinases (PDGFRα and PDGFRβ, Axl, RYK, EGFR, EphA2 and 10, IGF1R) as key targets of imatinib mesylate in HOS cells. In these cells, the phosphorylation status of these receptors was significantly decreased by imatinib mesylate, PDGFRα being particularly affected. We then studied the expression of PDGFRα in the various cell lines studied and confirmed its expression in human, mouse and rat osteosarcoma cells (Figure 7C). To confirm the functional activity of PDGFR in human and murine osteosarcoma cells, the PDGF-BB induced signaling was investigated (Figure 8). PDGF-BB induced in human (MG-63, HOS) and murine (POS-1, MOS-J) a rapid phosphorylation of PDGRα and PDFGRβ, as well as the downstream signaling pathways AKT and ERK1/2. The PDGF-BB induced phosphorylation cascades were markedly inhibited by 25 µM of imatinib mesylate confirming that both receptors represent functional targets in osteosarcoma (Figure 8).

## Discussion

Whereas metastasis is clearly the lethal process in osteosarcoma patients, the initial therapeutic response to chemotherapeutic drugs is a key aspect of the therapeutic care because it predicts the chance that growth of metastases is inhibited. Indeed, the current strategy for treatment of high-grade osteosarcoma is based on neo-adjuvant chemotherapy, delayed en-bloc wide resection, and adjuvant chemotherapy adapted to the histologic profile assessed on tumour tissue removed during surgery. The initial therapeutic response to the first line of chemotherapy will then condition the following therapeutic lines. Imatinib mesylate is a promising therapeutic drug targeting a large panel of tyrosine kinase proteins but also having an effect upon on tyrosine kinase targets such as quinine oxidoreductase and members of carbonic anhydrase family of metalloenzymes [21]–[23]. In this context, we investigated its effect on osteosarcoma cell lines *in vitro* and on tumour growth *in vivo*. Imatinib mesylate exerts anti-proliferative activities in human, mouse and rat osteosarcoma cell lines by affecting cell cycle and inducing caspase-dependent cell death and cell migration. Murine syngenic models of osteosarcoma have been used to study the efficacy of this therapeutic agent on the primary tumour growth at the bone site. The data revealed the inhibitory activity of imatinib mesylate in undifferentiated- and mixed osteoblastic−/osteolytic forms of osteosarcoma, in “preventive” and “curative” approaches. Finally, PDGFRα, Axl, PDGFRβ, RYK, EGFR, EphA2, EphA10 and IGF1-R are key targets of imatinib mesylate in osteosarcoma.

Osteosarcomas originate from connective tissues and thus derive from mesoderm. It has been suggested that osteosarcoma, chondrosarcoma and Ewing’s sarcoma originate from multipotent cells called mesenchymal stem cells able to differentiate into fibroblasts, osteoblasts, chondrocytes, adipocytes etc [1], [24]–[26]. Osteoblasts and their precursors are cellular targets of imatininb mesylate. Indeed, Indeed, imatinib mesylate inhibits osteoblast proliferation and activity in particular through its inhibitory activity on PDGFRβ [14]–[16]. We obtained similar findings with stimulation of calcified matrix deposition by osteoblasts correlating with modulation of differentiation markers (cbfa1, osteocalcine, bone sialoprotein) in the presence of low doses of imatinib mesylate (until 5 µM) while higher doses exert opposite effect with a strong inhibition of mineral deposit (Figure S4). Such marked effects of imatinib mesylate on osteoblastic lineage strengthen the therapeutic interest of imatinib mesylate for osteosarcoma. Ten years ago, McGary et al demonstrated that imatinib mesylate inhibits PDGF-mediated growth and leads to osteosarcoma cell apoptosis *in vitro* by selective inhibition of the PDGFR [27]. The effectiveness of the drug was confirmed in murine immunodeficient model of osteosarcoma as revealed by the follow-up of tumour-associated osteolysis by radiography [28]. Interestingly, expression of PDGF receptors and their ligands has been investigated in tissue micro arrays prepared from 54 osteosarcoma patients. Immunohistochemical analyses showed frequent expression of PDGFRα and PDGFRβ (around 80%) and their ligands with a correlation with lower event-free survival for PDGFRα that was not observed for PDGFRβ (28). In this context, the patient PDGFR status may be used as a prognostic marker in osteosarcoma and may serve to define imatinib mesylate therapy. Unfortunately, a phase II study of imatinib mesylate in children with solid tumours demonstrated little of no activity as a single agent in children with refractory osteosarcoma (10 cases evaluated) [29]. However, there was no information on the tyrosine kinase status of patients initially enrolled. Our data revealed that among the decreased phosphorylated receptors, PDGFRα appears as the most sensitive target of imatinib mesylate in osteosarcoma. In addition, while all cell lines were sensitive to this drug, PDGFRα was expressed by all cell lines in contrast to PDGFRβ. Our data are in agreement with the data obtained recently by Kitagawa et al [30]. These authors studied the specificities of various approved tyrosine kinase inhibitors including imatinib mesylate, by activity-based kinase profiling using a panel of 310 human recombinant active kinases. They revealed that imatinib mesylate targets were in rank order of IC50 values: PDFRα, PDFRβ, Discoidin Domain Receptor 2 (DDR2), DDR1, KIT, lymphocyte-specific protein tyrosine kinase (LCK), yes-related novel PTK B (LYN B), LYN A and ABL [30].

Our study demonstrated the inhibition of a set of tyrosine kinase receptors induced by imatinib mesylate (Figure 7). Ten years ago, from 600 cDNA microarray experiments of human osteosarcoma cell lines, five genes (Axl, TGFA, COLL7A1, WNT5A, and MKK6) have been identified and were associated with adherence, motility, and/or invasiveness of cancer cells [31]. More recently, using phosphoproteomic screening, Rettew et al identified twelve receptor tyrosine kinases that were phosphorylated in two metastatic human osteosarcoma cells (143B, LM7) [32]. In this extensive screening, these authors identified Axl, EphB2, FGFR2, IGF-1R and Ret as specific activated receptor tyrosine kinases and they demonstrated using functional inhibition approaches (neutralizing antibodies, antisense-mediated knockdown or small molecule inhibitors) that those specific receptors promote the in vitro behavior of metastatic osteosarcoma cell lines [32]. Axl appears to be expressed in most osteosarcoma tissues and its knockdown inhibits proliferation and induces apoptosis of human osteosarcoma cells [33], its expression predicting the clinical outcome of patients [34].

Human osteosarcoma also expressed numerous other targets of imatinib mesylate. Among them, cKit is one of the candidates [35]. No correlation was found between c-kit expression and overall or disease-free survival however c-kit-positive tumours exhibited lower necrosis post-chemotherapy [36]. Similarly the expression of EphA2 receptors is increased in osteosarcoma and modulates activation of the mitogenic signalling pathway and consequently may be involved in the oncogenic process [36]. In contrast to ephrinB ligands and EphB receptors which regulate the migration, attachment and spreading of mesenchymal stem cells, and constitute a bidirectional signaling between osteoclasts and osteoblasts activating osteoclasts, EphA2 and its ligands act as a “coupling inhibitor” [37]. Indeed, EphA2 reverse signaling into osteoclasts enhances osteoclastogenesis and suppresses osteoblastic bone formation. Consequently, EphA2 system may contribute to the pathogenesis of osteosarcoma by modulating the communications between tumour cells and their microenvironment [37]. Mass spectrometry experiments comparing human osteosarcoma cell lines and human primary osteoblasts identified 156 surface proteins significantly upregulated on osteosarcoma cells [38]. Among these proteins, EphA2 receptor 2 was the most abundant surface protein on cancer cells and was expressed in a majority of human osteosarcoma samples [38]. EphA10 may contribute to the oncologic process but its role remains under investigation [39]. EGFR and IGF1R have been also identified as therapeutic targets of tyrosine kinase inhibitors [40], [41]. RYK is a tyrosine receptor interacting with Wnt signalling [42] which strongly contributes to the dialog between tumour cells and their stromal environment, especially in osteosarcoma [43], [44].

The present data underline the potential therapeutic interest of imatinib mesylate in osteosarcoma. In light of these data and the literature, clinical investigations are absolutely required to evaluate its efficacy according the expression profile of tyrosine kinases before to conclude on the absence of effectiveness of imatinib mesylate in osteosarcoma patients. The therapeutic interest of imatinib mesylate in osteosarcoma then remains an open debate.

## Supporting Information

Figure S1 **Imatinib mesylate inhibits osteosarcoma cell divisions in a dose-dependent manner.** Human MG63, mouse MOS-J and rat OSRGA osteosarcoma cells were cultured in the presence or absence of increasing doses of imatinib mesylate. Phase-contrast photos were taken every 10 minutes for 72 hours and the number of cell mitosis manually scored in a time-dependent manner.
(TIF)

Figure S2 **Imatinib mesylate induces osteosarcoma cell death in a dose-dependent manner.** A kinetic of human (MG63), mouse (MOS-J) and rat (OSRGA) osteosarcoma cell death was analyzed by time-lapse microscopy in the presence or the absence of increasing doses of imatinib mesylate. The number of cell death was manually scored every 10 minutes until 72 hours.
(TIF)

Figure S3 **Effect of Imatinib mesylate on the osteosarcoma cell migration.** Osteosarcoma cell monolayers were damaged by scraping with a micropipette tip then incubated for 24 hours in the presence of 4 µg/mL mitomycin with or without imatinib mesylate (10–40 µg/mL). The extent of cell migration into the wounded area was analyzed by comparing microphotographs after 0 and 24 hours.
(TIF)

Figure S4 **Imatinib mesylate exhibits a dual effect on osteoblast differentiation.** Human mesenchymal stem cells (hMSC) were isolated, cultured, differentiated in osteoblasts and characterized according the technique described by Lavenus et al [45]. hMSC were cultured in the presence or absence of increasing concentrations of imatinib mesylate for 21 days and their ability to form mineralized matrix *in vitro* was revealed by alizarin red staining (A). Osteogenic makers [Runx2 (B), Osteocalcine (C), Bone Sialo Protein (D)] were followed by quantitative PCR. Imatinib mesylate exhibits a dual effect on osteoblast differentiation and acts as a pro-osteogenic factor until 5 µM and anti-osteogenic drug for higher concentrations.
(TIF)
