# Supplementary figures and images for: Imatinib Mesylate Exerts Anti-Proliferative Effects on Osteosarcoma Cells and Inhibits the Tumour Growth in Immunocompetent Murine Models
Source: PLoS One. 2014 Mar 5;9(3):e90795. doi: 10.1371/journal.pone.0090795 (PMC3944320; doi:10.1371/journal.pone.0090795)

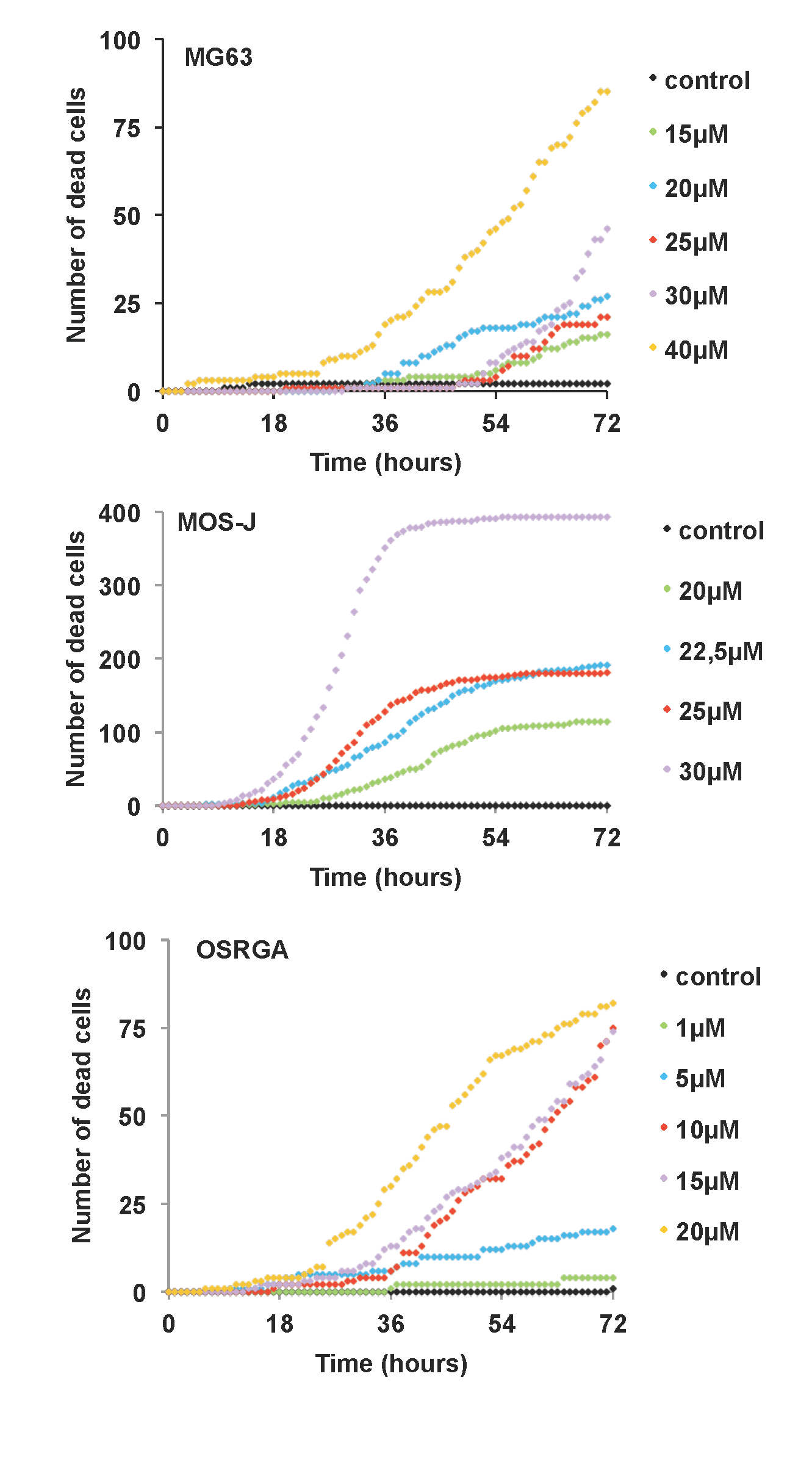

Supplement: Figure S2 — Imatinib mesylate induces osteosarcoma cell death in a dose-dependent manner. A kinetic of human (MG63), mouse (MOS-J) and rat (OSRGA) osteosarcoma cell death was analyzed by time-lapse microscopy in the presence or the absence of increasing doses of imatinib mesylate. The number of cell death was manually scored every 10 minutes until 72 hours. (TIF) [file pone.0090795.s002.tif]

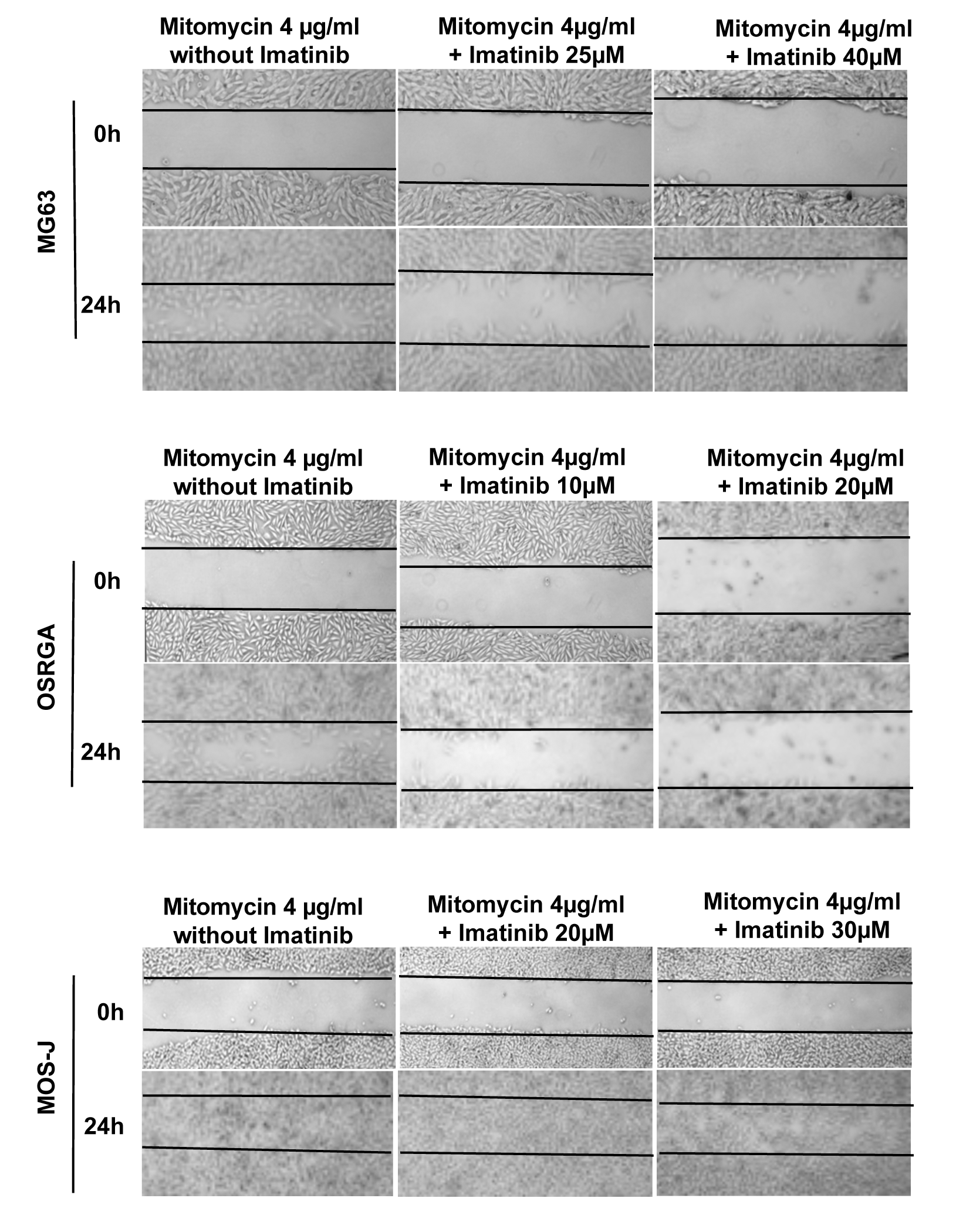

Supplement: Figure S3 — Effect of Imatinib mesylate on the osteosarcoma cell migration. Osteosarcoma cell monolayers were damaged by scraping with a micropipette tip then incubated for 24 hours in the presence of 4 µg/mL mitomycin with or without imatinib mesylate (10–40 µg/mL). The extent of cell migration into the wounded area was analyzed by comparing microphotographs after 0 and 24 hours. (TIF) [file pone.0090795.s003.tif]

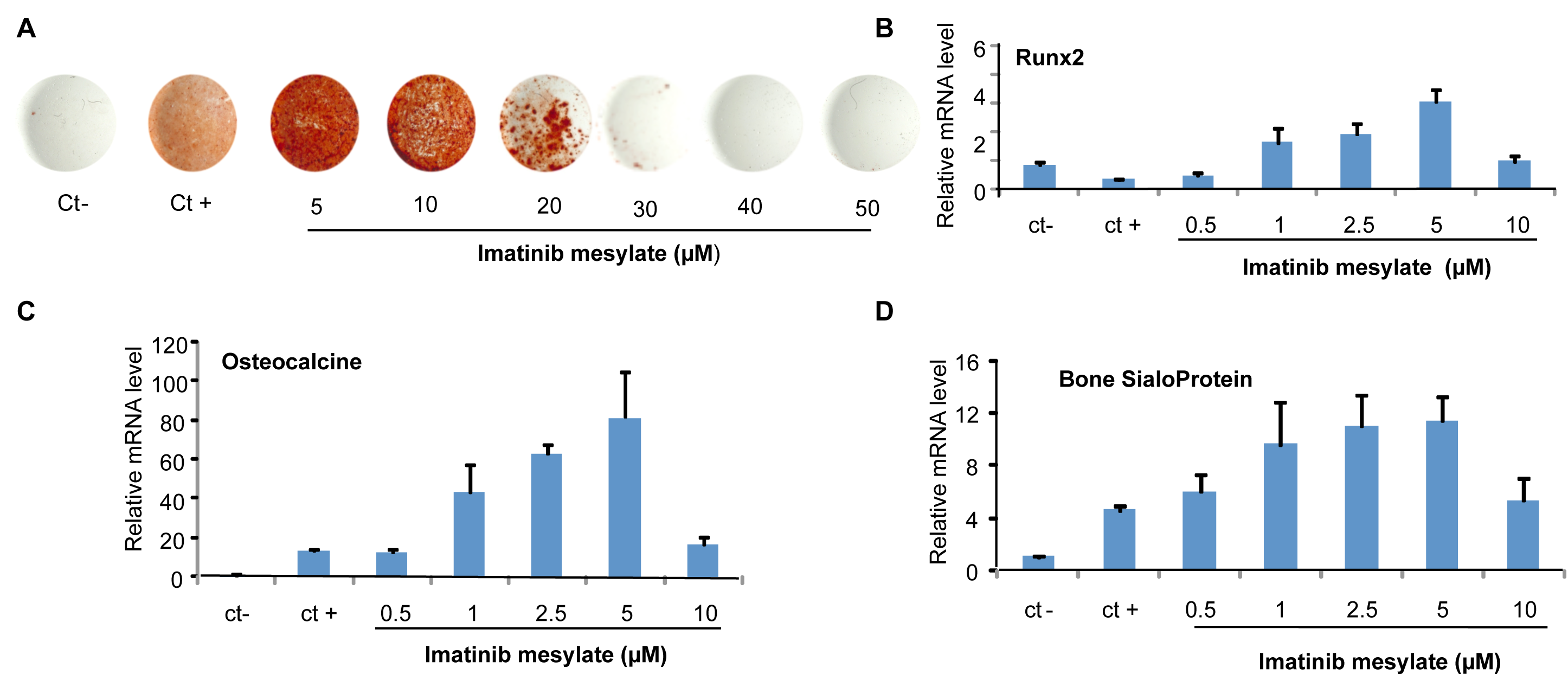

Supplement: Figure S4 — Imatinib mesylate exhibits a dual effect on osteoblast differentiation. Human mesenchymal stem cells (hMSC) were isolated, cultured, differentiated in osteoblasts and characterized according the technique described by Lavenus et al [45]. hMSC were cultured in the presence or absence of increasing concentrations of imatinib mesylate for 21 days and their ability to form mineralized matrix in vitro was revealed by alizarin red staining (A). Osteogenic makers [Runx2 (B), Osteocalcine (C), Bone Sialo Protein (D)] were followed by quantitative PCR. Imatinib mesylate exhibits a dual effect on osteoblast differentiation and acts as a pro-osteogenic factor until 5 µM and anti-osteogenic drug for higher concentrations. (TIF) [file pone.0090795.s004.tif]
